# Blood Lead Is a Predictor of Homocysteine Levels in a Population-Based Study of Older Adults

**DOI:** 10.1289/ehp.7369

**Published:** 2004-09-07

**Authors:** Jyme H. Schafer, Thomas A. Glass, Joseph Bressler, Andrew C. Todd, Brian S. Schwartz

**Affiliations:** ^1^Department of Environmental Health Sciences, Division of Occupational and Environmental Health, Johns Hopkins University Bloomberg School of Public Health, Baltimore, Maryland, USA; ^2^Department of Medicine, Johns Hopkins University, Baltimore, Maryland, USA; ^3^Department of Epidemiology, Johns Hopkins University Bloomberg School of Public Health, Baltimore, Maryland, USA; ^4^Department of Environmental Health Sciences, Division of Toxicological Sciences, Johns Hopkins University Bloomberg School of Public Health, Baltimore, Maryland, USA; ^5^Department of Neurology, Johns Hopkins University, Baltimore, Maryland, USA; ^6^Department of Neurotoxicology, Kennedy Krieger Institute, Baltimore, Maryland, USA; ^7^Department of Community and Preventive Medicine, Mount Sinai School of Medicine, New York, New York, USA

**Keywords:** blood lead, cross-sectional study, homocysteine, tibia lead

## Abstract

Lead and homocysteine are both associated with cardiovascular disease and cognitive dysfunction. We evaluated the relations among blood lead, tibia lead, and homocysteine levels by cross-sectional analysis of data among subjects in the Baltimore Memory Study, a longitudinal study of 1,140 randomly selected residents in Baltimore, Maryland, who were 50–70 years of age. Tibia lead was measured by ^109^Cd K-shell X-ray fluorescence. The subject population had a mean ± SD age of 59.3 ± 5.9 years and was 66.0% female, 53.9% white, and 41.4% black or African American. Mean ± SD blood lead, tibia lead, and homocysteine levels were 3.5 ± 2.4 μg/dL, 18.9 ± 12.5 μg/g, and 10.0 ± 4.1 μmol/L, respectively. In unadjusted analysis, blood lead and homocysteine were moderately correlated (Pearson’s *r* = 0.27, *p* < 0.01). After adjustment for age, sex, race/ethnicity, educational level, tobacco and alcohol consumption, and body mass index using multiple linear regression, results revealed that homocysteine levels increased 0.35 μmol/L per 1.0 μg/dL increase in blood lead (*p* < 0.01). The relations of blood lead with homocysteine levels did not differ in subgroups distinguished by age, sex, or race/ethnicity. Tibia lead was modestly correlated with blood lead (Pearson’s *r* = 0.12, *p* < 0.01) but was not associated with homocysteine levels. To our knowledge, these are the first data to reveal an association between blood lead and homocysteine. These results suggest that homocysteine could be a mechanism that underlies the effects of lead on the cardiovascular and central nervous systems, possibly offering new targets for intervention to prevent the long-term consequences of lead exposure.

As a result of centuries of human use, lead is omnipresent in the environment. Commercial use of this substance continues even though its toxic effects have been recognized since ancient times (Nriagu 1983), and more recent studies report health effects associated with lower and lower lead doses (Canfield et al. 2003; Glenn et al. 2003; Nash et al. 2003; Schwartz et al. 2000). Lead is not rapidly cleared from the body; the biologic residence time of lead in blood is measured in days, whereas the biologic residence time of lead in bone is on the order of years to decades (Hu et al. 1998). In occupational and general population samples, low blood lead levels have been associated with increased blood pressure and elevated risk of hypertension, effects that may be progressive (Cheng et al. 2001; Glenn et al. 2003; Nash et al. 2003); increased circulatory and cardiovascular mortality (Lustberg and Silbergeld 2002); and progressive declines in cognitive function over time, even years after cessation of occupational exposure (Schwartz et al. 2000, 2002, in press). One of the key remaining problems in the research of lead toxicity is that the mechanisms for these effects are not well understood.

Interestingly, homocysteine is also associated with cardiovascular disease and cognitive dysfunction (Dufouil et al. 2003; Homocysteine Collaboration 2002). Homocysteine is an independent risk factor for vaso-occlusive disease; elevated levels of homocysteine increase the risk of heart disease, stroke, and peripheral vascular disease and, perhaps through vascular mechanisms, cognitive dysfunction (Bautista et al. 2002; Dufouil et al. 2003; Homocysteine Collaboration 2002; Prins et al. 2002; Ravaglia et al. 2003; Wald et al. 2002). Vascular damage by homocysteine may occur through impaired vascular endothelial and smooth muscle cell function (Rodrigo et al. 2003). The mechanisms of this impairment may involve inhibition of nitric oxide synthesis, increased oxidative stress, proliferation of vascular smooth muscle cells, and altered elasticity of the vascular wall (Rodrigo et al. 2003).

Despite the similarities in these health effects, the relation of homocysteine and lead dose has not been previously examined. Herein, we report associations of blood lead, tibia lead, and homocysteine in a population-based study of persons 50–70 years of age in Baltimore, Maryland. Participants were selected from the general population, and most are without occupational lead exposure.

## Materials and Methods

### Study design.

The Baltimore Memory Study, one of the National Institutes of Health’s disparities initiative grants, is a multilevel cohort study of risk factors for cognitive decline in Baltimore city residents of targeted neighborhoods. The methods are described elsewhere (Schwartz et al. 2004). The selected neighborhoods were chosen to provide areas with a broad range of socioeconomic status and large numbers of both whites and African Americans. A cross-sectional analysis of first-visit data was performed.

### Subject selection and recruitment.

Sampling and recruitment have been previously described (Schwartz et al. 2004). In brief, individual dwellings in the study area were linked to telephone numbers, and households with telephones were randomly selected for recruitment. Eligibility was then determined on 2,351 subjects (50–70 years of age, living at selected household, lived in Baltimore at least 5 years), and of these subjects, 60.8% were scheduled for an enrollment visit. Of the 1,403 scheduled for an appointment, 1,140 (81.3%) were enrolled and subsequently tested. The study was approved by the Committee for Human Research of the Johns Hopkins Bloomberg School of Public Health. All participants provided written, informed consent before testing and were paid $50 for their participation.

### Data collection.

All data were collected at the study clinic by trained research assistants. A structured interview included the following information: demographics, socioeconomic status (household income, household assets, occupational status, and educational attainment), medical history, smoking and alcohol history, and lead history. First and second visits were conducted between May 2001 and September 2002, and October 2002 and February 2004, respectively. A trained phlebotomist drew a 10-mL blood specimen into a red-top (no anticoagulant) tube, which was clotted, centrifuged, and stored at –20°C within 1 hr. Samples were transported to the Johns Hopkins Bloomberg School of Public Health and stored at –70°C. Serum homocysteine was measured by a commercial laboratory using fluorescence polarization immunoassay (Abbott AxSYM, Abbott Park, IL). The coefficients of variation for the quality control samples for three concentration levels were 3.64% (low range), 2.24% (mid range), and 2.32% (high range). Fasting was not requested of the subjects because study visits were scheduled at all times of the day for logistical reasons. The variability between fasting and nonfasting samples is not likely to exaggerate the association but instead would dampen it. Conditions that cause short-term fluctuations in homocysteine levels, such as protein intake, are not likely to be related to whole-blood lead levels. Homocysteine was measured from a sample obtained at the first study visit in most subjects; however, 254 subjects provided plasma only at the first visit so had serum obtained at the second visit for homocysteine measurement. Lead was measured in the metals laboratory of the Kennedy Krieger Institute (Baltimore, MD) from the first study visit whole-blood specimen using anodic stripping voltammetry (Schwartz et al. 2004). Tibia lead concentration was measured at the second study visit by ^109^Cd-induced K-shell X-ray fluorescence using previously reported methods (Todd 2000; Todd and McNeill 1993; Todd et al. 1992, 2000). In this population of older adults without occupational lead exposure, tibia lead, which has a biologic residence time of 25–30 years, should not have changed appreciably between the first and second study visits.

### Statistical methods.

The main objectives of this analysis were *a*) to evaluate relations of blood lead and tibia lead levels with homocysteine, controlling for age, race/ethnicity, sex, and other potential confounding variables; and *b*) to evaluate whether these relations were modified by age, sex, or race/ethnicity. Of the 1,140 persons enrolled at the first visit, 78 subjects were missing homocysteine values; 10 were missing blood lead values; 7 missing information on alcohol consumption; 6 missing body mass index (BMI); and 1 each was missing information on education and tobacco use. A total of 1,022 (89.6%) subjects completed the second study visit, so 82 participants were missing tibia lead data. Thus, in analyses with blood lead and tibia lead, 1,037 and 955 subjects were included, respectively. Subjects with missing homocysteine data were not statistically different regarding blood lead, age, or race/ethnicity. To minimize the influence of large tibia lead values, tibia lead was log transformed before use in models. Negative values for tibia lead were converted to 0.1 before log transformation. Associations with tibia lead were also examined nonparametrically, using a percentile transformation for tibia lead; results did not differ from those using the log-transformed tibia lead and are not reported.

We used multiple linear regression to examine the relations of blood lead with homocysteine levels, controlling for covariates. Models were first constructed including known homocysteine covariates (e.g., age, sex, and race/ethnicity); then, other potential confounding variables were included in a forward stepwise fashion. Variables were retained in the final models if they were associated with homocysteine levels or significantly influenced the relation of blood or tibia lead with homocysteine (changed the lead coefficient by more than 10%). The final model included age, race/ethnicity, sex, educational level (four categories based on reported years of education and information on graduate equivalency diploma, training for trades, and additional educational certificates), alcohol (four categories based on the number of alcoholic drinks per month, with a drink being defined as one beer, one glass of wine, one wine cooler, one cocktail, or one shot of liquor), smoking (four categories based on the number of cigarettes smoked per day), and BMI (kilograms per square meter). We evaluated effect modification by including cross-product terms in the model (e.g., to evaluate effect modification by race/ethnicity, a cross-product of race/ethnicity and blood lead was included in the model). All statistical analyses were performed using Stata version 8.0 (Stata Corporation, College Station, TX). We checked final models for the assumptions of linear regression and model fit using influence diagnostic procedures, examination of residuals, and residual–residual plots.

## Results

**Figure 1:**
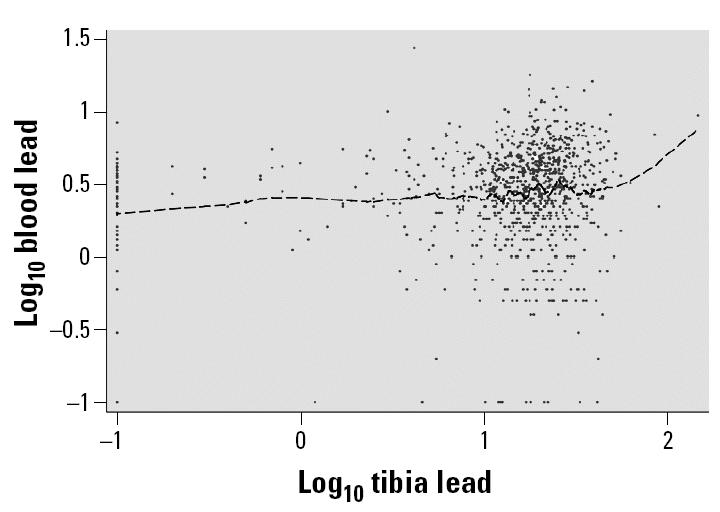
Crude relation of log_10_-transformed blood lead levels and log_10_-transformed tibia lead levels for 955 participants in the Baltimore Memory Study. Dashed line represents a locally weighted smoothing fit (lowess bandwidth, 0.10) (Cleveland 1979).

Study subjects were 66.0% female, 41.4% non-Hispanic black/African American, and 53.9% non-Hispanic white or white/Native American and had a mean (range) age of 59.3 (49–71) years. The mean ± SD blood lead and homocysteine levels were 3.5 ± 2.4 μg/dL and 10.0 (4.1) μmol/L, respectively (Table 1). Subjects had a wide range of educational levels, smoking habits, and alcohol habits; in unadjusted analysis, these were differentially associated with blood lead and homocysteine levels, evaluated in quartiles (Tables 1 and 2). Using blood lead and homocysteine as continuous measures, in unadjusted analysis, these were moderately correlated, with Pearson’s *r* = 0.27 (*p* < 0.01). Blood lead and tibia lead levels were only modestly correlated [Pearson’s *r* = 0.11, for the log transformed data (Figure 1), and *r* = 0.12 for the untransformed data; both *p*-values < 0.01].

## Discussion

**Figure 2:**
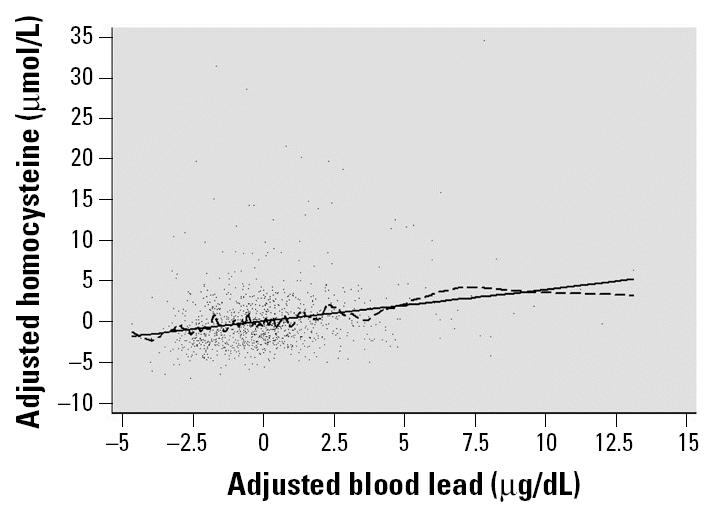
Smoothed plot of residuals of blood lead and homocysteine levels, controlling for covariates. Values were adjusted for age, sex, race/ethnicity, BMI, educational level, and tobacco and alcohol use. The three data points with blood lead concentrations > 15 μg/dL have been excluded from the plot (but not from the regression model) so that the portion of the plot with the most data could be more clearly visualized. The solid line is predicted linear fit, and the dashed line is from a locally weighted smoothing fit (lowess bandwidth, 0.05) (Cleveland 1979).

To our knowledge, this is the first study to examine relations of blood lead and tibia lead with homocysteine levels. We observed a significant association between blood lead and homocysteine in an older, community-dwelling, adult, population-based sample in a major U.S. urban area after controlling for age, sex, race/ethnicity, alcohol intake, cigarette smoking, educational level, and BMI. As previously observed, sex, age, and smoking a pack or more per day were predictors of homocysteine levels (Jacques et al. 2001). Blood lead may influence homocysteine levels at very low dose levels (Figure 2). Among study subjects, blood lead levels were generally < 15 μg/dL, as expected in the general population. The study thus provides evidence of an association at low blood lead levels, but we were unable to characterize the association at higher blood lead levels. Although tibia lead was modestly associated with blood lead levels, it was neither a predictor of homocysteine levels nor a confounder of its relation with blood lead. Tibia lead levels were obtained at the second study visit. Subjects who did not complete the second study visit were more likely to be African American (52.4 vs. 40.4%) and were slightly younger (58.5 vs. 59.4 years of age) compared with subjects who completed the visit, but there was no difference in blood lead levels. We do not believe these small differences account for the contrasting associations of blood and tibia lead levels with homocysteine. Tibia lead, a measure of cortical bone lead, is generally a less important source of blood lead levels than is lead in trabecular bone, but is a good estimate of cumulative lead dose (Hu et al. 1998). The data suggest that bioavailable lead (i.e., blood lead) was a more important predictor of homocysteine levels than was cumulative lead dose (i.e., tibia lead).

Lead and homocysteine are both associated with an increased risk of cardiovascular disease and, possibly through vascular system mechanisms, central nervous system disease. In epidemiologic studies of central nervous system disease and cardiovascular outcomes, it is interesting to note that study results for lead parallel those for homocysteine. For example, in an occupational cohort of men with previous lead exposure (on average 18 years prior), the systolic blood pressure increased on average 0.64 mm Hg (SE = 0.25) for every SD increase in blood lead at baseline (Glenn et al. 2003). In a recent meta-analysis using data from 30 prospective or retrospective studies, a 25% lower homocysteine level (~ 3 μmol/L) was associated with an approximately 11% [odds ratio (OR) = 0.89; 95% confidence interval (CI), 0.83–0.96] lower ischemic heart disease risk and a 19% (OR = 0.81; 95% CI, 0.69–0.95) lower stroke risk (Homocysteine Collaboration 2002). Another meta-analysis of 20 prospective studies found that for an increase in serum homocysteine of 5 μmol/L, the OR for ischemic heart disease was increased (OR = 1.32; 95% CI, 1.19–1.45), as was the OR for stroke (OR = 1.59; 95% CI, 1.29–1.96) (Wald et al. 2002). Other studies support the similarities between the cardiovascular effects of lead and homocysteine (Bautista et al. 2002; Cheng et al. 2001; Kopp et al. 1988; Moller and Kristensen 1992; Nash et al. 2003; Pocock et al. 1988).

Both lead and homocysteine also have similar associations with central nervous system outcomes [Agency for Toxic Substances and Disease Registry (ATSDR) 1999; Balbus-Kornfeld et al. 1995; Dufouil et al. 2003; Prins et al. 2002; Ravaglia et al. 2003; Schwartz et al. 2000, 2001]. There are suggestions that cognitive function is negatively affected by elevated levels of either lead or homocysteine. In a population-based study of 1,077 subjects, neurobehavioral test outcomes were lower in the upper quintile of homocysteine levels compared with the lower four quintiles (Prins et al. 2002). The mean ± SD homocysteine level of this population was 11.5 ± 4.1 μmol/L, with the medians of the quintiles being 7.57, 9.12, 10.54, 12.47, and 16.34 μmol/L, respectively. Comparison of the mean adjusted difference in test scores for subjects in the homocysteine upper quintile versus lower quintiles (looked at dichotomously) revealed a decrement in psychomotor speed (–0.26; 95% CI, ^–^0.37–0.14), memory function (–0.13; 95% CI, ^–^0.27–0.01), and global cognitive function (–0.20; 95% CI, ^–^0.30–0.11). In a longitudinal study of 1,241 subjects (61–73 years of age), homocysteine levels > 15 μmol/L conferred 2.8 greater odds (*p* < 0.05) of cognitive decline compared with those subjects whose levels were < 10 μmol/L (mean homocysteine level in this study was 12.2 μmol/L) (Dufouil et al. 2003). For lead, two recent longitudinal studies of occupational cohorts with current and/or past lead exposure were associated with longitudinal decline in cognitive function (Schwartz et al. 2000, in press). One of the studies consisted of former organolead workers (mean age, 55.6 years) and used an extensive neurobehavioral test battery (Schwartz et al. 2000). In this study, an increase of 15.7 μg/g of peak tibia lead (using back-extrapolation, the estimated tibia lead level at the end of employment in lead) was equivalent in its effects on annual test decline to 5 more years of age at baseline for six neurobehavioral tests of verbal memory and learning, executive abilities, and manual dexterity. Although blood lead, but not tibia lead, was associated with homocysteine levels, in this cross-sectional study we cannot be certain whether this means that recent lead exposure, mobilization of lead from bone, or both, are the likely source of lead that explains the association. With the growing evidence that lead may cause progressive elevations in blood pressure and declines in cognitive function over time (Glenn et al. 2003; Schwartz et al. 2000, 2001, in press), this newly observed association between lead and homocysteine may offer new possibilities for preventive intervention.

Several targets that lead could be acting upon could explain this association. Lead can interact with proteins, particularly those with a sulfhydryl group (Goering 1993). An example of this occurrence is the inhibition of the δ-aminolevulinic acid dehydratase (ALAD) enzyme in the heme-synthesis pathway. ALAD is an octameric metalloenzyme that contains zinc in the activated state (Simons 1995). The active site for zinc binding contains two cysteine residues. There is competitive inhibition between lead and zinc, with the ratio of the affinity of lead to zinc at the metal-binding site being about 25:1 for the 1-1 ALAD phenotype (Simons 1995). Such sulfhydryl binding by lead could be one mechanism that could account for the observed lead–homocysteine relation. In the metabolism of methionine, homocysteine can be remethylated by two different pathways or undergo transsulferation to cysteine (Ueland and Refsum 1989). In the transsulferation pathway there is a unique heme-containing enzyme, cystathionine β-synthase, that catalyses a pyridoxal 5′-phosphate–dependent condensation of serine and homocysteine to give cystathionine (Banerjee et al. 2003). Work in the elucidation of the structure of cystathionine β-synthase has revealed two sulfhydryl groups contained within the heme-binding site (Meier et al. 2001). Furthermore, homocysteine itself contains a sulfhydryl group, so if lead has an affinity for this sulfhydryl group, the metabolism of homocysteine could be directly inhibited, leading to an accumulation of homocysteine.

It has been unclear whether homocysteine is a causative agent or only a marker of disease. In 1962, homocystinuria in mentally retarded children was discovered as an inborn error of metabolism (Carson and Neill 1962; Gerritsen et al. 1962). In 1964, cystathionine β-synthetase deficiency was demonstrated as a cause of this disorder (Mudd et al. 1964). The natural history of cystathionine β-synthetase deficiency includes a 50% chance of a vascular event (stroke, myocardial infarction, peripheral arterial or venous thrombosis) by 30 years of age (Yap 2003). Recent experimental evidence suggests homocysteine to be a causal agent. Experimentation has shown isolated hyper-homocysteinemia to be atherogenic in cystathionine β-synthetase and apolipoprotein-E double knock-out mice (Wang et al. 2003). Additionally, homocysteine has been shown to stimulate the expression and secretion of biologically active monocyte chemoattractant protein-1 (MCP-1) and interleukin-8 (IL-8) in human monocytes (Zeng et al. 2003), two chemokines that are thought to be important to the development of atherosclerotic plaques.

In conclusion, blood lead was found to be associated with homocysteine levels in a large, general population sample. Although causality cannot be determined from cross-sectional data, it is interesting to consider the possibility that this relation of lead and homocysteine could explain one of the mechanisms of the influence of lead on the central nervous and cardiovascular systems. Whether lead elevates homocysteine through enzyme inhibition, as earlier suggested, or conversely, whether homocysteine elevates lead because of intravascular binding [homocysteine has a structure similar to dimercaptosuccinic acid (DMSA) and penicillamine, compounds that are known to bind lead], it is evident that the association exists at very low blood lead levels, and if the former mechanism is operative, supports a biologic effect of lead at low levels. This knowledge may offer new targets for prevention of the progressive health effects of lead.

## Figures and Tables

**Table 1:** Demographic characteristics for the inclusive study population and lead quartile subsets, Baltimore Memory Study, 2001–2002.

		Blood lead quartile				
Characteristic	Total (*n* = 1,037)	Quartile 1 (*n* = 241)	Quartile 2 (*n* = 271)	Quartile 3 (*n* = 262)	Quartile 4 (*n* = 263)	*p*-Value*^a^*
Blood lead level [mean (range) μg/dL]	3.5 (0.1–27.3)	1.1 (0.1–1.9)	2.5 (2.0–3.0)	3.8 (3.1–4.4)	6.5 (4.5–27.3)	
Homocysteine [mean ± SD (μmol/L)]	10.0 ± 4.1	9.2 ± 3.4	9.3 ± 3.5	9.7 ± 3.6	11.7 ± 5.1	< 0.001
Age [mean ± SD (years)]	59.3 ± 5.9	59.3 ± 5.9	59.3 ± 5.8	59.0 ± 5.9	59.8 ± 6.2	0.52
Sex (% female)	66.0	83.0	72.3	63.7	46.0	< 0.001
Race/ethnicity (%)						0.73
Non-Hispanic black/African American	41.4	44.4	39.9	38.9	42.6	
Non-Hispanic white or white/Native American	53.9	49.4	55.7	56.1	54.0	
African American mixed race/ethnicity	2.7	2.9	3.0	3.1	1.9	
Asian, Hawaiian, Native American, or other	2.0	3.3	1.4	1.9	1.5	
BMI [mean ± SD (kg/m^2^)]	29.8 ± 6.9	31.5 ± 7.8	30.7 ± 7.2	28.8 ± 6.4	28.3 ± 5.4	< 0.001
Current cigarette use (%)						0.008
None	79.8	85.9	80.8	82.4	70.7	
< Half pack per day	5.6	3.7	7.8	3.5	7.2	
Half pack to < 1 pack per day	7.6	4.2	5.9	8.0	12.2	
≥ 1 pack per day	7.0	6.2	5.5	6.1	9.9	
Alcoholic beverage use (%)						< 0.001
None	40.6	49.9	43.9	38.5	30.8	
< 4 per month	15.1	17.4	15.9	13.4	13.7	
4–8 per month	12.8	11.2	15.1	14.5	10.3	
> 8 per month	31.5	21.6	25.1	33.6	45.2	
Education level (%)						0.9
< High school or trade school	10.2	10.4	9.2	8.4	12.9	
Completed high school or trade school	41.7	41.5	43.2	41.2	40.7	
Some college or associate degree	5.9	6.2	6.3	6.1	4.9	
≥ College degree	42.2	41.9	41.3	44.3	41.5	

a *p*-Value from chi-square test for categorical variables or for continuous variables analysis of variance *F*-test for linear trend across quartiles.

**Table 2:** Demographic characteristics of study subjects by homocysteine quartiles, Baltimore Memory Study, 2001–2002.

	Homocysteine quartile				
Characteristic	Quartile 1 (*n* = 245)	Quartile 2 (*n* = 269)	Quartile 3 (*n* = 259)	Quartile 4 (*n* = 264)	*p*-Value*^a^*
Homocysteine [mean (range) μmol/L]	6.6 (4.4–7.5)	8.3 (7.6–9.0)	10.0 (9.1–11.2)	15.0 (11.3–48.6)	
Blood lead level [mean ± SD (μg/dL)]	2.8 ± 1.6	3.2 ± 2.4	3.7 ± 2.1	4.4 ± 2.8	< 0.001
Age [mean ± SD (years)]	57.9 ± 5.5	59.1 ± 5.8	60.0 ± 6.0	60.3 ± 6.2	< 0.001
Sex (% female)	87.8	69.1	59.5	48.9	< 0.001
Race/ethnicity (%)					0.001
Non-Hispanic black/African American	39.2	36.1	42.9	47.4	
Non-Hispanic white or white/Native American	51.8	62.4	53.6	47.3	
African American/mixed race/ethnicity	5.7	0.4	2.7	2.3	
Asian, Hawaiian, Native American or other	3.3	1.1	0.8	3.0	
BMI [mean ± SD (kg/m^2^)]	29.2 ± 6.6	29.6 ± 7.0	30.2 ± 6.8	30.2 ± 7.0	0.27
Current cigarette use (%)					0.001
None	84.5	85.1	79.1	70.8	
< Half pack per day	6.1	4.5	4.2	7.6	
Half pack to less than 1 pack per day	5.3	5.6	9.7	9.9	
≥ 1 pack per day	4.1	4.8	7.0	11.7	
Alcoholic beverage use (%)					0.007
None	44.5	39.0	40.9	38.3	
< 4 per month	15.9	15.6	14.7	14.0	
4–8 per month	18.0	13.0	12.0	8.7	
> 8 per month	21.6	32.4	32.4	39.0	
Education level (%)					0.002
< High school or trade school	10.2	7.1	10.4	13.2	
Completed high school or trade school	39.6	37.2	40.9	48.9	
Some college or associates degree	6.5	3.7	8.1	5.3	
≥ College degree	43.7	52.0	40.6	32.6	

a *p*-Value from chi-square for categorical variables or for continuous variables analysis of variance *F*-test for linear trend across quartiles.

**Table 3:** Predictors*^a^* of homocysteine levels in subjects with complete data (*n* = 1,037), Baltimore Memory Study, 2001–2002.

	Total (*n* = 1,037)		Female (*n*= 684)		Male (*n* = 353)	
	β(SE β)	*p*-Value	β(SE β)	*p*-Value	β(SE β)	*p*-Value
Blood lead (μg/dL)	0.35 (0.05)	< 0.001	0.24 (0.07)	0.001	0.43 (0.08)	< 0.001
Age (years)	0.09 (0.02)	< 0.001	0.14 (0.02)	< 0.001	–0.02 (0.04)	0.61
Female	–1.46 (0.27)	< 0.001	—	—	—	—
BMI (kg/m^2^)	0.05 (0.02)	0.004	0.05 (0.02)	0.02	0.09 (0.04)	0.03
Current cigarette use*^b^*						
< Half pack per day	1.01 (0.53)	0.06	0.78 (0.62)	0.20	1.30 (0.96)	0.18
Half pack to < 1 pack per day	1.53 (0.47)	0.001	2.05 (0.56)	< 0.001	0.80 (0.84)	0.35
≥1 packs per day	2.29 (0.49)	< 0.001	2.12 (0.60)	< 0.001	2.11 (0.83)	0.01

a Adjusted for variables in table as well as race/ethnicity (four categories), educational level (four categories), and alcohol consumption (none, < 4 per month, 4–8 per month, > 8 per month).

b Reference group is subjects with no current use.
